# Influence of vitamin D supplementation on muscle strength and exercise capacity in South African schoolchildren: secondary outcomes from a randomised controlled trial (ViDiKids)

**DOI:** 10.1136/bmjsem-2024-002019

**Published:** 2024-09-26

**Authors:** Keren Middelkoop, Lisa Micklesfield, Stephanie Hemmings, Neil Walker, Justine Stewart, David A Jolliffe, Amy E Mendham, Jonathan C Y Tang, Cyrus Cooper, Nicholas C Harvey, Robert J Wilkinson, Adrian R Martineau

**Affiliations:** 1 Desmond Tutu HIV Centre, Institute of Infectious Diseases & Molecular Medicine, University of Cape Town, Observatory, South Africa; 2 Department of Medicine, University of Cape Town, Observatory, South Africa; 3 Health through Physical Activity, Lifestyle and Sport Research Centre (HPALS), Division of Physiological Sciences, Department of Human Biology, Faculty of Health Sciences, University of Cape Town, Cape Town, South Africa; 4 SAMRC/Wits Developmental Pathways for Health Research Unit, Department of Paediatrics, Faculty of Health Sciences, University of the Witwatersrand, Johannesburg, South Africa; 5 School of Sport and Health Sciences, University of Brighton, Eastbourne, UK; 6 Wolfson Institute of Population Health, Faculty of Medicine and Dentistry, Queen Mary University of London, London, UK; 7 Blizard Institute, Faculty of Medicine and Dentistry, Queen Mary University of London, London, UK; 8 Riverland Academy of Clinical Excellence, Riverland Mallee Coorong Local Health Network, South Australia Health, South Australia, Australia; 9 Norwich Medical School, University of East Anglia, Norwich, UK; 10 Departments of Laboratory Medicine, Clinical Biochemistry and Departments of Diabetes and Endocrinology, Norfolk and Norwich University Hospital NHS Foundation Trust, Norwich, UK; 11 MRC Lifecourse Epidemiology Centre, University of Southampton, Southampton, UK; 12 NIHR Southampton Biomedical Research Centre, University of Southampton, and University Hospital Southampton NHS Foundation Trust, Southampton, UK; 13 Centre for Infectious Diseases Research in Africa, Institute of Infectious Disease and Molecular Medicine, University of Cape Town, Observatory, South Africa; 14 The Francis Crick Institute, London, UK; 15 Imperial College London, London, UK

**Keywords:** Children's health and exercise, Nutrition, Fitness testing

## Abstract

**Objective:**

To determine whether vitamin D supplementation influences grip strength, explosive leg power, cardiorespiratory fitness and risk of exercise-induced bronchoconstriction (EIB) in South African schoolchildren.

**Methods:**

Substudy (n=450) in Cape Town schoolchildren aged 8–11 years nested within a phase 3 randomised placebo-controlled trial (ViDiKids). The intervention was weekly oral doses of 10 000 IU vitamin D_3_ (n=228) or placebo (n=222) for 3 years. Outcome measures were serum 25-hydroxyvitamin D_3_ (25(OH)D_3_) concentrations, grip strength, standing long jump distance, peak oxygen uptake (VO_2peak_, determined using 20 m multistage shuttle run tests) and the proportion of children with EIB, measured at end-study.

**Results:**

64.7% of participants had serum 25(OH)D_3_concentrations <75 nmol/L at baseline. At 3-year follow-up, children randomised to vitamin D versus placebo had higher mean serum 25(OH)D_3_ concentrations (97.6 vs 58.8 nmol/L, respectively; adjusted mean difference 39.9 nmol/L, 95% CI 36.1 to 43.6). However, this was not associated with end-study differences in grip strength, standing long jump distance, VO_2peak_ or risk of EIB.

**Conclusion:**

A 3-year course of weekly oral supplementation with 10 000 IU vitamin D_3_ elevated serum 25(OH)D_3_ concentrations in South African schoolchildren but did not influence muscle strength, exercise capacity or risk of EIB.

**WHAT IS ALREADY KNOWN ON THIS TOPIC**

- Observational studies have reported that vitamin D deficiency is associated with reduced muscle strength and peak oxygen uptake and increased risk of exercise-induced bronchoconstriction (EIB) in children. Randomised controlled trials (RCTs) of vitamin D supplementation to improve children’s muscle strength and cardiorespiratory fitness have yielded conflicting results.

**WHAT THIS STUDY ADDS**

- This RCT, conducted in South African schoolchildren aged 8–11 years at baseline, found that a 3-year course of weekly oral supplementation with 10 000 IU vitamin D_3_ improved vitamin D status but had no effect on grip strength, standing long jump distance, peak oxygen uptake or risk of EIB.

**HOW THIS STUDY MIGHT AFFECT RESEARCH, PRACTICE OR POLICY**

- Taken together with null results from another phase 3 RCT of vitamin D supplementation conducted in Mongolian children, our findings do not suggest that weekly oral vitamin D supplementation exerts clinically significant effects on muscle strength or exercise tolerance in schoolchildren in whom rickets have been excluded.

## Introduction

 Muscle strength and exercise tolerance positively correlate with physical and mental health during childhood and are associated with reduced risk of cardiometabolic disease in adulthood.^1^,^4^ Vitamin D is essential for the normal development and function of the cardiorespiratory system and skeletal muscle.^5^,^7^ Deficiency in this fat-soluble micronutrient—indicated by low circulating concentrations of 25-hydroxyvitamin D (25(OH)D)—is widespread among children in both higher-income and lower-income countries.^8^,^11^ Observational studies have linked vitamin D deficiency in children and adolescents to reduced muscle strength and cardiorespiratory fitness^12 13^ and increased risk of exercise-induced bronchoconstriction (EIB),^14^ which may limit exercise capacity. Numerous randomised controlled trials (RCTs) in adults have revealed positive effects of vitamin D supplementation on muscle strength and power, with meta-analyses reporting a modest benefit overall.^15^ However, analogous studies in children are less numerous and have yielded inconsistent outcomes. For example, an RCT of vitamin D supplementation conducted in Tunisia in vitamin D deficient male soccer players aged 8–15 years reported improvements in long jump distance, sprint and shuttle run outcomes^16^ while a UK study in girls aged 12–14 years showed that vitamin D supplements enhanced movement efficiency, with indications of better jumping velocity and grip among participants randomised to intervention.^17^ However, RCTs in children and adolescents living in Denmark,^18 19^ the USA,^20^ Israel^21^ and Lebanon^22^ have not found significant effects of vitamin D supplementation on grip strength, leg press strength or swimming performance. No such trials have yet been conducted in Africa; moreover, there is a lack of large, multicentre trials examining the impact of prolonged (greater than 1 year) vitamin D supplementation on muscle strength, cardiorespiratory fitness and risk of EIB in children living in any setting.

Given these limitations of the existing evidence base, we took the opportunity to generate new data in this area during the conduct of a phase 3 RCT of weekly vitamin D supplementation involving a total of 1682 children attending primary schools in Cape Town, South Africa. The primary objective was to determine whether this intervention would decrease the risk of incident tuberculosis (TB) infection, as indicated by the conversion of an interferon-gamma release assay from negative at baseline to positive at 3-year follow-up; null findings for this outcome have been reported elsewhere.^23^ The current paper reports findings of a substudy nested within the main trial, which tested the hypotheses that vitamin D supplementation would improve grip strength, standing long jump distance, peak oxygen uptake (VO_2peak_) and reduce risk of EIB in a subset of 450 participants aged 8–11 years at baseline.

## Methods

### Trial design, setting, approvals and registration

We conducted a multicentre phase 3 individually randomised placebo-controlled trial in 23 government schools in Cape Town, South Africa, as previously described.^23^,^25^ The primary outcome was the acquisition of latent TB infection; the current manuscript reports the effects of the intervention on prespecified secondary outcomes relating to muscle strength and cardiorespiratory outcomes measured at 3-year follow-up in a subset of 450 participants who took part in a nested substudy. The trial was sponsored by Queen Mary University of London and registered on the South African National Clinical Trials Register (DOH-27-0916-5527) and ClinicalTrials.gov (ref NCT02880982).

### Participants

Inclusion criteria for the main trial were enrolment in grades 1–4 at a participating school; aged 6–11 years at screening and written informed assent/consent to participate in the main trial provided by children and their parent/legal guardian, respectively. Exclusion criteria for the main trial were a history of previous latent TB infection, active TB disease or any chronic illness other than asthma (including known or suspected HIV infection) prior to enrolment; use of any regular medication other than asthma medication; use of vitamin D supplements at a dose of more than 400 IU/day in the month before enrolment; plans to move away from study area within 3 years of enrolment; inability to swallow a placebo soft gel capsule with ease and clinical evidence of rickets or a positive QuantiFERON-TB Gold Plus (QFT-Plus) assay result at screening. Additional inclusion criteria for the substudy were enrolment in grade 4 at a participating school; additional exclusion criteria for the substudy were the presence of an injury or disability limiting the ability to run with maximum effort and the inability to perform spirometry at baseline. Trial staff who determined whether subjects were eligible for inclusion in the trial were unaware of which group the next subject would be allocated to, that is, allocation was concealed.

### Enrolment and baseline assessments

Parents or legal guardians were invited to provide written informed consent for their child to participate in the main trial during a home visit, unless their child was eligible for the substudy, in which case they were invited to provide written informed consent for their child to participate in both the main trial and the substudy until a total of 450 substudy participants were randomised. If parents/legal guardians consented, they were asked to provide details of their child’s dietary intake of foods containing vitamin D and calcium in the previous month, which were captured on an electronic case report form as previously described.^24^ Their children were then invited to provide written assent to participate in the main trial±the substudy (if eligible) at a school-based visit. If they agreed, a clinically trained member of the study team screened them for symptoms and signs of rickets. For all participants, a blood sample was taken for a QFT-Plus assay (Qiagen, Hilden, Germany) and separation and storage of serum for determination of 25(OH)D concentrations as described below. Participants were reviewed when baseline QFT-Plus results were available. Those with a positive QFT-Plus result were excluded from the trial and screened for active TB. Those with an indeterminate QFT-Plus result were excluded from the trial without screening for active TB. Those with a negative QFT-Plus result were deemed eligible to participate and underwent measurement of weight (using a digital floor scale, Charder Medical) and height (using a portable HM200P stadiometer, Charder Medical). For substudy participants, grip strength was measured as described elsewhere^26^ using a portable dynamometer (Takei Digital Grip Strength Dynamometer, Model T.K.K.5401); the best of two readings for the dominant hand were recorded, except where injury precluded measurement, where strength of the non-dominant hand was measured. Standing long jump distance was measured as described elsewhere^27^ using a DiCUNO measuring tape, with the best of two readings recorded. Validity and reliability of the Takei Digital Grip Strength Dynamometer for measurement of grip strength, and of the standing long jump for measurement of lower body muscular power have both been demonstrated in schoolchildren studied in other settings.^28 29^ Spirometry was performed according to European Respiratory Society/American Thoracic Society standards^30^ using a portable spirometer (SpiroUSB, Carefusion, San Diego, USA). A 20 m multistage shuttle run test was then conducted using freely available recorded instruction (Shuttle run bleep test, www.bleeptests.com, accessed on 26 March 2024) until volitional exhaustion. Two lines were marked 20 m apart and an audible ‘bleep’ signalled to participants the speed required to run between them. The number of completed laps was recorded and used to derive VO_2peak_ as described below. Spirometry was repeated 3–5 min after completion of the shuttle run test to detect EIB.

### Randomisation and blinding

Eligible and assenting children whose parents consented to their participation in the trial were individually randomised to receive a weekly capsule containing vitamin D_3_ or placebo for 3 years, with a one-to-one allocation ratio and randomisation stratified by school of attendance, as previously described.^23^ Full details are supplied in online supplemental file 1. The randomisation list was computer generated by a statistician (Prof Richard Hooper, Queen Mary University of London) prior to the start of recruitment. This list was held by the Data Monitoring Committee during the conduct of the trial. Concealment of allocation was achieved by ensuring that no trial staff (including those who assessed eligibility) had access to the trial randomisation list. All participants and their parents/guardians, and all trial personnel, including principal investigators and all those who had contact with study participants, including those who assessed outcomes, were blinded to participant allocation during the conduct of the trial.

### Intervention

Study medication comprised a 3-year course of weekly soft gel capsules manufactured by the Tishcon Corporation (Westbury, New York, USA), containing either 0.25 mg (10 000 international units) cholecalciferol (vitamin D_3_) in olive oil (intervention arm) or olive oil without any vitamin D_3_ content (placebo arm). A slightly lower dose was employed in this trial as compared with the sister trial in Mongolia (which investigated the effects of administering 14 000 IU vitamin D_3_ weekly),^31^ as we anticipated that South African participants’ baseline vitamin D status would be higher than in Mongolia and that accordingly a lower dose of vitamin D would be needed to optimise vitamin D status of South African participants, randomised to the intervention arm. Active and placebo capsules had identical appearance and taste. Vitamin D_3_ content of active capsules was confirmed by the manufacturer using high-performance liquid chromatography (LC). Capsules were taken under direct observation of study staff during the school term time. During the summer holidays (8 weeks), packs containing eight doses of study medication were provided for administration by parents, together with a participant diary. Following shorter school holidays (≤4 weeks) and/or if participants missed one or more doses of study medication during term time, up to four ‘catch-up’ doses were administered at the first weekly visit attended following the missed dose(s). During the initial national lockdown for COVID-19 in South Africa (27 March 2020–1^t^ May 2020), participants did not receive any study medication. During subsequent school closures due to COVID-19, two rounds of 8-week holiday packs were provided to participants, which were sufficient to cover their requirements until schools reopened.

### Outcomes

The primary outcome for the main trial, reported elsewhere,^23^ was the QuantiFERON-TB Gold Plus result at the manufacturer-recommended 0.35 IU/mL threshold at the end of the study. The following secondary outcomes were assessed for substudy participants at 3-year follow-up: maximal grip strength (kg), standing long jump distance (cm), VO_2peak_ (mL/kg/min, derived from shuttle run performance) and the proportion of participants with EIB, defined as a ≥10% decrease in forced expiratory volume in 1 s after completion versus before the shuttle run test.^32^

### Laboratory assessments

Biochemical analyses were performed at the Bioanalytical Facility, University of East Anglia (Norwich, UK) according to manufacturers’ instructions and under Good Clinical and Laboratory Practice conditions. Serum concentrations of 25(OH)D_3_ were measured using LC tandem mass spectrometry (LC-MS/MS) as previously described.^10^ 25(OH)D_3_ was calibrated using standard reference material SRM972a from the National Institute of Science and Technology (NIST), and the assay showed linearity between 0 and 200 nmol/L. The interassay/intra-assay coefficient of variation across the assay range was ≤9%, and the lower limit of quantification was 0.1 nmol/L. The assay showed <6% accuracy bias against NIST reference method on the vitamin D external quality assessment (DEQAS) scheme (http://www.deqas.org/; accessed on 18 March 2024). QFT-Plus assays were performed by the Bio Analytical Research Corporation South Africa (Johannesburg, South Africa) according to the manufacturer’s instructions.

### Sample size

The sample size for the main trial was predicated on the power to detect an effect of the intervention on the primary outcome (the proportion of children with a positive QFT-Plus assay result at 3-year follow-up), as previously described.^23^ Sample size for the substudy was predicated on power to detect an effect of the intervention on bone mineral content: assuming 29% loss to follow-up at year 3, we calculated that enrolment of 450 participants would provide 88% power to detect a difference of 0.35 SDs between arms for mean bone mineral content, at either site investigated at the 5% significance level.

### Statistical analyses

Statistical analyses were performed by using Stata software (V.17.0; StataCorp) according to intention to treat. Values of p<0.05 were considered to be statistically significant. VO_2peak_ was calculated from the number of completed laps on shuttle run tests using a published formula.^33^ Effects of treatment on continuous outcomes measured at 3-year follow-up were analysed using multilevel mixed models with random effects of school and individual and with the treatment effect at baseline constrained to be zero to reflect the randomisation, where applicable. Proportions of children with EIB at 3-year follow-up were analysed using a mixed-effects logistic regression model, with treatment allocation as the sole fixed effect and school attended as a random intercept and adjustment for presence versus absence of EIB at baseline. Prespecified subgroup analyses were conducted to determine whether the effect of vitamin D supplementation was modified by sex (male vs female), baseline deseasonalised 25(OH)D_3_ concentration, calculated using a sinusoidal model as previously described^34^ (<75 vs ≥75 nmol/L) and estimated calcium intake (< vs ≥ median value of 466 mg/day, calculated as previously described).^24^ These were performed by repeating efficacy analyses with the inclusion of an interaction term between allocation (to vitamin D vs placebo) and each posited effect-modifier with the presentation of the p value associated with this interaction term. Given the number of potential effect modifiers and secondary outcome measures, these analyses are considered exploratory. Interim safety assessments, where independent data monitoring committee (IDMC) members reviewed accumulating serious adverse event data, were performed at 6-monthly intervals. At each review, the IDMC recommended continuation of the trial. No interim efficacy analysis was performed.

### Patient and public involvement

The ViDiKids trial was designed in consultation with the local Community Advisory Board (CAB)—a committee of local community stakeholders. Plans for enrolment and implementation of the study protocol were discussed with the school principals, staff and parent bodies prior to study initiation. Collaborative relationships with participating schools meant that the staff assisted in addressing study challenges, especially those that arose as a result of the COVID-19 pandemic. Dissemination of study results to the CAB, the school staff and parent bodies by a team including a staff member fluent in the local language is ongoing.

### Equity, diversity and inclusion statement

This trial recruited a group of participants who are under-represented in research (schoolchildren in South Africa). Participation was open to children of any gender, race/ethnicity/culture and socioeconomic level. The author team is gender-balanced and nationality-balanced and includes junior researchers and perspectives from multiple disciplines. Subgroup analyses were conducted to detect gender differences in response to vitamin D supplementation.

## Results

### Participants

**Figure 1:**
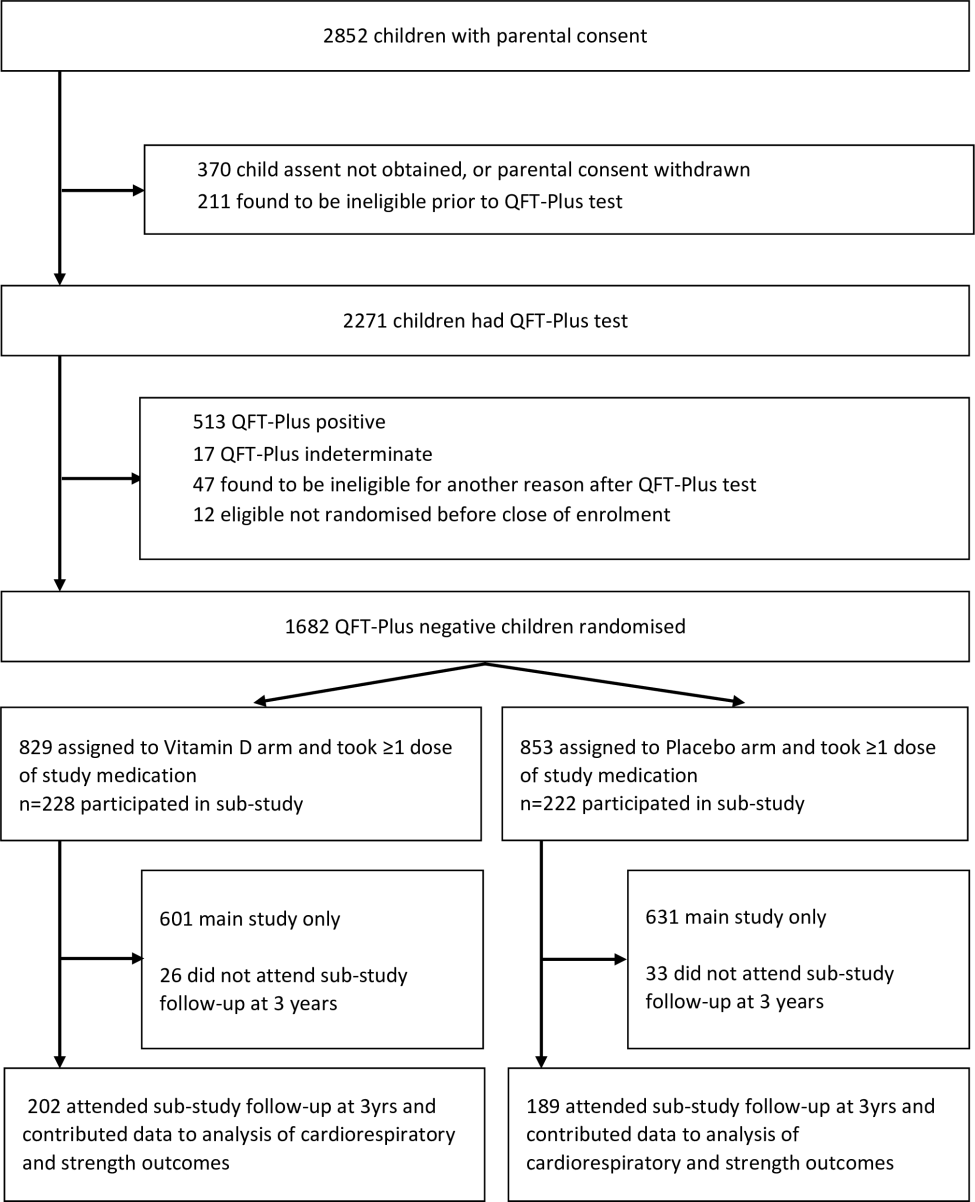
Participant flow diagram. QFT-Plus, QuantiFERON-TB Gold Plus.

2852 children were screened for eligibility to participate in the main trial from March 2017 to March 2019, of whom 2271 underwent QFT testing: 1682 (74.1%) QFT-negative children were randomly assigned to receive vitamin D_3_ (829 participants) or placebo (853 participants) as previously described.^23^ 450/1682 (26.8%) participants in the main trial also participated in the substudy, of whom 228 vs 222 participants were allocated to the vitamin D versus placebo arms, respectively (figure 1). Table 1 shows the baseline characteristics of participants contributing data to analyses of cardiorespiratory and strength outcomes: overall, mean age was 10.1 years, 52.0% were female and mean deseasonalised baseline serum 25(OH)D concentration was 70.0 nmol/L (SD 13.5 nmol/L). These and other baseline characteristics were well balanced between substudy participants randomised to vitamin D versus placebo. Of 450 randomised substudy participants, 391 (86.9%) attended the substudy follow-up visit at 3 years postrandomisation and contributed data to analyses presented here. Mean serum 25(OH)D_3_ concentration at 3-year follow-up was higher in participants randomised to vitamin D versus placebo (97.6 vs 58.8 nmol/L, respectively: mean difference 39.9 nmol/L, 95% CI 36.1 to 43.6).

**Table 1:** Participants’ baseline characteristics, overall and by allocation

		Overall(n=450)	Vitamin D arm(n=228)	Placebo arm (n=222)
Mean age, years (SD) (range)		10.1 (0.7)(8.2 to 11.9)	10.2 (0.7)(8.2 to 11.9)	10.0 (0.6)(8.5 to 11.9)
Sex, n (%)	Female	234 (52.0)	116 (50.9)	118 (53.2)
	Male	216 (48.0)	112 (49.1)	104 (46.8)
Ethnic origin*	Xhosa, n (%)	424 (96.4)	214 (96.0)	210 (96.8)
	Other, n (%)	16 (3.6)	9 (4.0)	7 (3.2)
Type of residence	Brick, n (%)	230 (51.1)	121 (53.1)	109 (49.1)
	Informal, n (%)	220 (48.9)	107 (46.9)	113 (50.9)
Parental education†	None, n (%)	-	-	-
	Primary school, n (%)	24 (5.3)	16 (7.0)	8 (3.6)
	Secondary school or higher, n (%)	426 (94.7)	212 (93.0)	214 (96.4)
Mean monthly household income, ZAR1000 (SD)		1.6 (2.3)	1.5 (2.7)	1.6 (1.9)
Household environmental tobacco smoke, n (%)		47 (10.4)	28 (12.3)	19 (8.6)
Mean maximal grip strength, kg (SD)‡		13.9 (3.4)	14.2 (3.6)	13.7 (3.1)
Mean standing long jump distance, cm (SD)‡		120.0 (18.4)	121.7 (18.5)	118.2 (18.1)
Mean VO_2peak_, mL/kg/min (SD)‡		40.5 (3.2)	40.5 (3.0)	40.5 (3.3)
Exercise-induced bronchospasm, proportion (%)‡		79/448 (17.6)	38/228 (16.7)	41/220 (18.6)
Mean serum 25(OH)D_3_, nmol/L (SD) (range)§		70.0 (13.5)(24.9–112.5)	70.4 (12.1)(43.2–101.3)	69.6 (14.9)(24.9–112.5)
Serum 25(OH)D_3_ categories, n (%)§	<25 nmol/L	1 (0.3)	0 (0.0)	1 (0.6)
	25–49.9 nmol/L	20 (5.5)	7 (3.7)	13 (7.4)
	50–74.9 nmol/L	214 (59.0)	114 (61.0)	100 (56.8)
	≥75 nmol/L	128 (35.3)	66 (35.3)	62 (35.2)

* Ethnicity data missing in 5 participants in both the vitamin D and placebo arms.

† Highest level of education of at least 1 parent.

‡ Data missing in 2 participants in the placebo arm.

§ Serum 25(OH)D_3_ concentrations deseasonalised; data missing in 41 participants in the vitamin D arm and 46 participants in the placebo arm.

25(OH)D_3_, 25-hydroxyvitamin D_3_; VO_2peak_, peak oxygen consumption; ZAR, South African Rand

### Outcomes

Allocation to vitamin D versus placebo did not influence grip strength or long jump distance, either overall or within subgroups defined by male versus female sex, baseline 25(OH)D concentration or calcium intake (table 2). No effect of allocation to vitamin D versus placebo was seen for mean VO_2peak_, either overall or by subgroup (table 3). Similarly, no effect of allocation was seen on the proportion of participants with EIB, either overall or by subgroup (table 4).

**Table 2:** Maximal grip strength and standing long jump distance at 3-year follow-up by allocation: overall and by subgroups

		Vitamin D: mean value, kg (SD) (n)	Placebo: mean value, kg (SD) (n)	Adjusted mean difference (95% CI)*	P value	P for interaction
Grip strength						
Overall		20.9 (5.1) (201)	20.6 (5.2) (188)	−0.19 (−0.94 to 0.55)	0.61	--
By sex	Male	22.0 (5.5) (96)	21.3 (5.9) (89)	0.04 (−1.24 to 1.32)	0.95	0.57
	Female	19.8 (4.4) (105)	20.0 (4.4) (99)	−0.38 (−1.22 to 0.46)	0.37	
By baseline 25(OH)D concentration†	<75 nmol/L	20.6 (4.7) (103)	21.1 (5.3) (96)	−0.51 (−1.58 to 0.56)	0.35	0.14
	≥75 nmol/L	21.7 (6.2) (60)	20.5 (5.3) (54)	0.74 (−0.64 to 2.12)	0.30	
By calcium intake	<median‡	20.9 (4.6) (93)	20.9 (5.5) (97)	−0.25 (−1.43 to 0.94)	0.68	0.65
	≥median‡	21.0 (5.6) (102)	20.5 (4.8) (88)	−0.12 (−1.02 to 0.78)	0.79	
		**Vitamin D: mean value, cm (SD) (n)**	**Placebo: mean value, cm (SD) (n)**	**Adjusted mean difference (95% CI)** *	**P value**	**P for interaction**
Long jump distance						
Overall		128.2 (22.1) (201)	122.1 (21.0) (188)	3.58 (−0.00 to 7.16)	0.0502	--
By sex	Male	140.5 (20.3) (96)	132.7 (20.2) (89)	5.89 (0.80 to 10.99)	0.02	0.32
	Female	117.0 (17.2) (105)	112.6 (17.0) (99)	2.58 (−1.47 to 6.62)	0.21	
By baseline 25(OH)D concentration†	<75 nmol/L	130.0 (24.4) (93)	124.7 (20.7) (97)	1.14 (−3.76 to 6.05)	0.65	0.62
	≥75 nmol/L	126.7 (19.3) (102)	119.9 (21.2) (88)	3.66 (−2.54 to 9.86)	0.25	
By calcium intake	<median‡	129.97 (24.39) (93)	124.70 (20.72) (97)	3.33 (−1.67 to 8.33)	0.19	0.98
	≥median‡	126.73 (19.35) (102)	119.86 (21.21) (88)	3.86 (−1.32 to 9.03)	0.14	

* Adjusted for baseline value and school of attendance.

† Serum 25(OH)D_3_ concentrations deseasonalised.

‡ Median value 466 mg/day.

25(OH)D_3_25-hydroxyvitamin D_3_

**Table 3:** VO_2peak_ at 3-year follow-up by allocation: overall and by subgroups

		Vitamin D arm: mean value, mL/kg/min (SD) (n)	Placebo arm: mean value, mL/kg/min (SD) (n)	Adjusted mean difference (95% CI)*	P value	P for interaction
Overall		37.2 (4.1) (200)	37.1 (4.1) (187)	0.24 (−0.15 to 0.63)	0.23	
By sex	Male	39.0 (3.2) (95)	39.2 (3.4) (89)	−0.02 (−0.56 to 0.53)	0.95	0.22
	Female	35.5 (4.1) (105)	35.2 (3.8) (98)	0.47 (−0.06 to 1.00)	0.08	
By baseline 25(OH)D concentration†	<25 nmol/L	36.6 (4.5) (103)	36.7 (3.9) (95)	0.50 (−0.06 to 1.06)	0.08	0.28
	≥25 nmol/L	37.7 (3.9) (59)	37.5 (4.2) (54)	−0.01 (−0.75 to 0.74)	0.98	
By calcium intake	<median‡	37.5 (3.7) (92)	37.4 (4.3) (97)	0.19 (−0.32 to 0.70)	0.47	0.87
	≥median‡	36.8 (4.5) (102)	36.6 (4.0) (87)	0.25 (−0.36 to 0.87)	0.42	

* Adjusted for baseline value and school of attendance.

† Serum 25(OH)D_3_ concentrations deseasonalised.

‡ Median value 466 mg/day.

25(OH)D_3_25(OH)D_3_, 25-hydroxyvitamin D_3_

**Table 4:** Proportion of participants with exercise-induced bronchoconstriction at 3-year follow-up by allocation: overall and by subgroup

		Vitamin D: proportion (%)	Placebo: proportion (%)	Adjusted OR (95% CI)*	P value	P for interaction
Overall		29/200 (14.50)	16/187 (8.56)	1.92 (0.99 to 3.73)	0.053	
By sex	Male	13/95 (13.68)	5/89 (5.62)	2.86 (0.95 to 8.61)	0.06	0.32
	Female	16/105 (15.24)	11/98 (11.22)	1.48 (0.65 to 3.41)	0.35	
By baseline 25(OH)D concentration†	<75 nmol/L	14/103 (13.59)	8/95 (8.42)	1.82 (0.70 to 4.72)	0.22	0.39
	≥75 nmol/L	7/59 (11.86)	2/54 (3.70)	3.49 (0.69 to 17.68)	0.13	
By calcium intake	<median‡	13/92 (14.13)	4/97 (4.12)	3.86 (1.17 to 12.70)	0.03	0.08
	≥median‡	14/102 (13.73)	12/87 (13.79)	1.04 (0.44 to 2.46)	0.93	

* Adjusted for school of attendance and presence versus absence of exercise-induced bronchoconstrictionn at baseline.

† Deseasonalised values.

‡ Median value 466 mg/day.

25(OH)D25-hydroxyvitamin D

## Discussion

We present findings of the first RCT investigating the effects of vitamin D supplementation on muscle strength and cardiorespiratory fitness to be conducted in Africa—and the first RCT conducted anywhere to test whether vitamin D supplementation influences the risk of EIB. Weekly oral administration of 10 000 IU vitamin D_3_ was effective in elevating circulating 25(OH)D concentrations; however, this was not associated with any statistically significant differences in any other outcome investigated. Our null findings for grip strength, long jump distance and VO_2peak_ are in keeping with those from a similarly designed phase 3 RCT conducted in Mongolia, where the effects of a 3-year course of 14 000 IU vitamin D/week were investigated.^3135^,^37^

Our study has several strengths. Weekly vitamin D supplementation allowed direct observation of capsule administration during term time, and this was reflected in the significant interarm difference in serum 25(OH)D concentrations observed at 3-year follow-up. 25(OH)D concentrations were measured using the gold-standard methodology (LC-MS/MS) in a laboratory that participated in a rigorous external quality assessment scheme (DEQAS). Low rates of loss to follow-up maximised our power to detect effects of the intervention, and we assessed a comprehensive range of outcomes relating to muscle strength, cardiorespiratory fitness and respiratory function.

Our study also has some limitations. Fewer than 6% of children had baseline 25(OH)D concentrations <50 nmol/L, limiting generalisability of our findings to settings where vitamin D deficiency is more common. Additionally, we draw attention to that fact that our findings relate specifically to the effects of weekly vitamin D supplementation. Daily administration of vitamin D has been reported to be more beneficial for prevention of acute respiratory infections than weekly administration,^38^ and the possibility that this may also be the case for musculoskeletal and cardiorespiratory outcomes cannot be excluded. Further trials comparing the effects of daily versus weekly supplementation would be needed to resolve this question definitively. We also acknowledge that the parameters investigated in the current study were secondary outcomes (although prespecified ones), and that multiple tests for statistical significance were performed; we would not, therefore, have been able to rule out type I error as an explanation for statistically significant findings, had these arisen. In the absence of validated equations to estimate VO_2peak_ from shuttle run performance in South African children, we used equations developed for East Asian children, in order to maximise comparability of our findings with those of a similarly designed RCT conducted in Mongolia.^31^ While we acknowledge that this may have introduced a degree of imprecision, randomisation will very likely have distributed this equally between intervention versus control arms, minimising any risk of bias.

In conclusion, we report that a weekly oral dose of 10 000 IU vitamin D_3_, administered to South African children for 3 years, was effective in elevating serum 25(OH)D concentrations but did not influence grip strength, long jump distance, VO_2peak_ or risk of EIB. Taken together with null results from a similarly designed phase 3 RCT conducted in Ulaanbaatar, Mongolia,^31^ our study does not suggest a role for weekly oral vitamin D supplementation to enhance muscle strength or exercise capacity in schoolchildren in whom rickets have been excluded.

## supplementary material

10.1136/bmjsem-2024-002019 online supplemental file 1

## Data Availability

Data are available on reasonable request.
